# Biological Activities of Gedunin—A Limonoid from the Meliaceae Family

**DOI:** 10.3390/molecules25030493

**Published:** 2020-01-23

**Authors:** Teresa M. Braga, Lídia Rocha, Tsz Yan Chung, Rita F. Oliveira, Cláudia Pinho, Ana I. Oliveira, Joaquim Morgado, Agostinho Cruz

**Affiliations:** 1 Centro de Investigação em Saúde e Ambiente, Escola Superior de Saúde, Instituto Politécnico do Porto, 4200-072 Porto, Portugal; lcar@ess.ipp.pt (L.R.); tycg@ess.ipp.pt (T.Y.C.); rfo@ess.ipp.pt (R.F.O.); clp@ess.ipp.pt (C.P.); aoliveira@ess.ipp.pt (A.I.O.); 2 Bio4Life4You, 4460-170 Porto, Portugal; jcmorgado@bio4life4you.com; 3 World Neem Organization, Mumbai 400101, India

**Keywords:** gedunin, limonoid, Meliaceae family, bioactive compound, biological activity

## Abstract

Gedunin is an important limonoid present in several genera of the Meliaceae family, mainly in seeds. Several biological activities have been attributed to gedunin, including antibacterial, insecticidal, antimalarial, antiallergic, anti-inflammatory, anticancer, and neuroprotective effects. The discovery of gedunin as a heat shock protein (Hsp) inhibitor represented a very important landmark for its application as a biological therapeutic agent. The current study is a critical literature review based on the several biological activities so far described for gedunin, its therapeutic effect on some human diseases, and future directions of research for this natural compound.

## 1. Introduction

**Figure 1:**
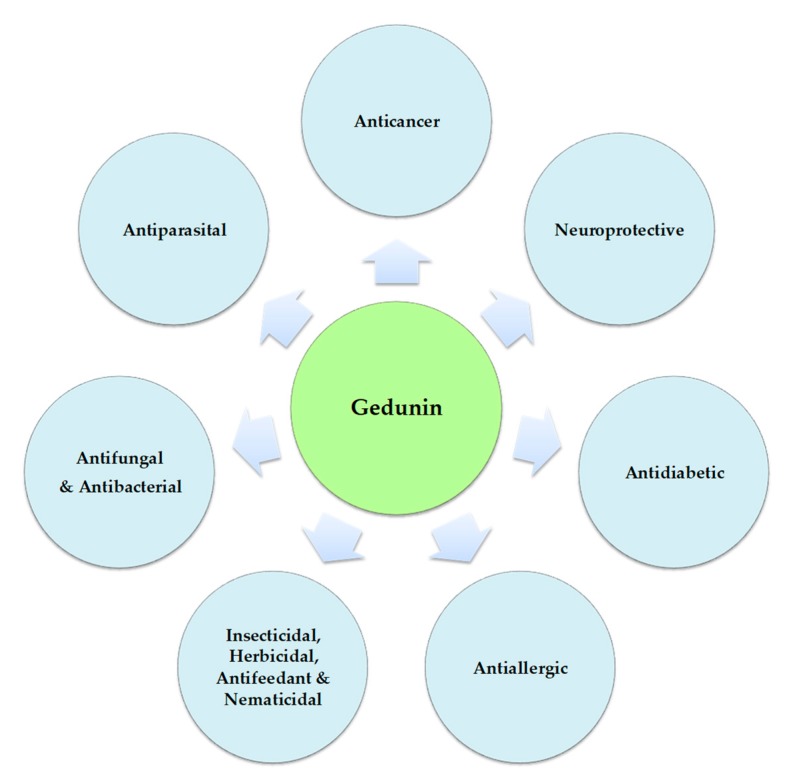
Biological activities of gedunin.

The World Health Organization (WHO) estimates the existence of 20,000 different medicinal plants in 91 different countries [1]. Throughout time, many civilizations have used different species of these plants for their potential to treat diseases. That is the case of many species of plants from the Meliaceae family, which are used in traditional medicine and also in pest control. This family, distributed in tropical and subtropical regions, includes more than 50 genera with about 1400 species [2]. Of the various phytochemical constituents isolated from different parts of plants in this family, limonoids are the most relevant. Limonoids are described as exhibiting a range of biological activities, with insecticidal, antifungal, antimalarial, antibacterial, antiviral, and anticancer effects [3]. Besides the Meliaceae family, limonoids are also found in the Rutaceae family and, less frequently, in the Cneoraceae family [4]. More than 300 limonoids have been isolated and they are described as more abundant and diverse in the Meliaceae family than in any other family [2]. An important limonoid, gedunin (and/or a number of its derivatives), has been reported in various genera of the Meliaceae family, e.g., *Azadirachta*, *Cabralea*, *Carapa*, *Cedrela*, *Chukrasia*, *Entandrophragm*, *Guarea*, *Trichilia*, and *Xylocarpus* [5,6,7]. Gedunin is abundant in the fruit epicarp of *Azadirachta indica* A. Juss. and has higher concentrations in young green fruits than in ripe fruits [8]. *A. indica* leaves presented gedunin concentrations lower than 0.1% and a residual presence elsewhere in the plant. Several biological activities have been attributed to gedunin, such as antibacterial, antifungal, antimalarial, insecticidal, antiallergic, anti-inflammatory, anticancer, and neuroprotective effects (Figure 1) [4,9,10,11].

The present review aims to provide a state-of-the-art analysis about gedunin, focusing in the several biological activities described and the importance of this natural product in the development of new therapeutics. This manuscript represents, to our knowledge, the first review about this important limonoid from the Meliaceae family.

## 2. Chemistry

**Figure 2:**
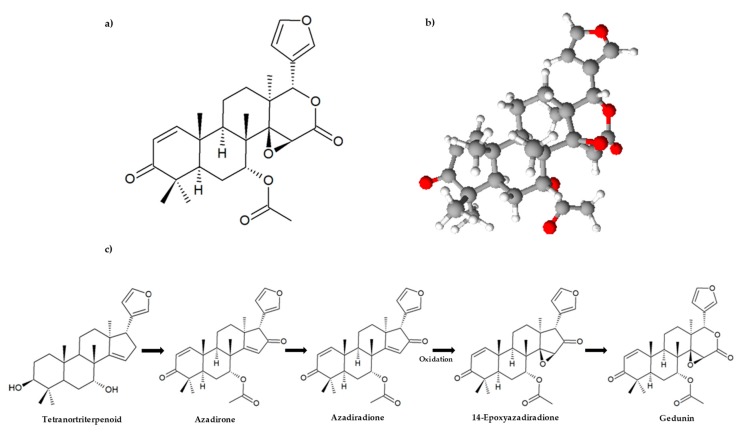
Chemical structure of gedunin: (**a**) Two-dimensional structural formula and (**b**) three-dimensional model; (**c**) Biosynthesis pathway of gedunin (adapted from Narender et al. [12] and Aarthy et al. [15]). ChemSketch software was used to create all chemical structures.

Structurally, limonoids are formed by loss of four terminal carbons of the side chain in the apotirucallane or apoeuphane skeleton and then cyclized to form the 17β-furan ring. This is why they are also known as tetranortriterpenoids [4], and classified according to which of the four rings (A, B, C and D), in the intact triterpene nucleus, was oxidized [12]. Gedunin is the most representative member of the ring d-seco class of limonoids. In this group, the δ-lactone in ring D derived from the azadirone class undergoes a ring oxidative expansion through a Baeyer–Villiger type reaction, having a 4,4,8-trimethyl-17-furanyl steroid skeleton [4,13,14]. The biosynthetic pathway leading to the formation of gedunin from a tetranortriterpenoid is presented in Figure 2c. Several steps from this pathway are still uncharacterized. However, Aarthy et al. [15] recently discovered, for the neem tree, the crucial role of the mevalonic acid (MVA) pathway as the only source of isoprene units for limonoid biosynthesis, and that the amino acid isoleucine and leucine biosynthetic pathways contribute to the building of the functional groups of limonoids. The molecular formula of gedunin is C_28_H_34_O_7_ (MW: 482.55 g/mol), and it was first isolated from the West African timber *Entandrophragma angolense* (Welw.) C. DC. and named by Akisanya and his co-authors in 1960 [16,17]. Later, in 1961, the same authors described some reactions of gedunin, which were explained by a structure similar to that proven for limonin [17]. The application of nuclear magnetic resonance (NMR), mass spectrometry (MS), and X-ray diffraction analysis contributed to gedunin characterization, its constitution, and its relative stereochemistry, using a dihydrogedun-3β-yl iodoacetate derivative [18,19,20,21]. These data confirm the chemical structure of gedunin presented in Figure 2a,b.

Gedunin, isolated from *Trichilia pallida* Sw., crystallizes in the orthorhombic space group *P2_1_2_1_2_1_*, with two independent molecules in the asymmetric unit (α-Gedunin and β-Gedunin) [6]. Its asymmetric unit arrangement is described as being spontaneous and exothermically produced in the steps of crystallization. The molecular electrostatic potential (MEP) map revealed that the negative potential sites are around electronegative atoms, and the positive potential sites around the hydrogen atoms. The interaction between conformers is strong (13.09 kcal/mol).

The total synthesis of gedunin has not yet been reached. Semi-synthetic derivatives of gedunin have been successfully produced by chemical modifications to the gedunin scaffold, but they presented lower biological activity than the original compound [22,23,24]. Recent studies have achieved a full construct of the elaborated ABC ring system by using a Robinson annulation reaction, which facilitated the installation of the 7-acetoxy functional group and completion of the ring [25,26], and revealed the first synthetic route to the BCD ring system of the unsaturated ring d-seco limonoids [27].

## 3. Biological Activities

### 3.1. Anticancer Activity

Gedunin has been reported as an anticancer agent, mainly as a cell proliferation inhibitor and apoptosis inductor. This section presents several anticancer studies using gedunin (Table 1) and its importance as an inhibitor of heat shock protein 90 (Hsp90) activity.

#### 3.1.1. Heat Shock Protein 90 Inhibition Activity

Hsp90, one of the most abundant proteins in cells, is an adenosine triphosphate (ATP)-dependent molecular chaperone and is induced when a cell suffers several environmental stresses, such as cold, heat, or lack of oxygen [46,47]. It is involved in the turnover, trafficking, and activity of various oncogenic proteins (usually referred as “client proteins”) including apoptotic factors, transcription factors, protein kinases, signaling proteins, and a number of oncoproteins. It has also been described to be associated to some neurodegenerative, vascular, and metabolic diseases. Recently, some plant-derived small molecules, such as gedunin, have been proved to exhibit inhibitory activity towards Hsp90. This was described for the first time by Lamb et al. [37], who discovered that gedunin exhibited antiproliferative activity by modulating Hsp90. The same authors later described gedunin as an androgen receptor (AR)-mediated signaling inhibitor and predicted that it also acted as an Hsp90 pathway inhibitor, promoting client protein degradation and the activation of heat shock factor 1 (HSF1) [38]. At that time, a new mechanism of action of Hsp90 inhibitor was proposed for gedunin, outside the N-terminal ATP-binding pocket. Gedunin was also verified as a disruptor of Hsp 90 co-chaperone encoded by the CDC37 gene (Cdc37)/Hsp90 interactions through modulation of Hsp90 [48]. Gedunin’s cytotoxic effect is due to its binding to p23 co-chaperone of Hsp90. This interaction inactivates Hsp90, which leads to destabilization of its client proteins. Computational modeling showed that gedunin forms hydrogen bond contacts with Thr 90 and Lys 95 at the C-terminal region of p23 and forms hydrophobic interactions with side chain of Ala 94 [30]. Recently, in order to better understand the likely mode of binding between gedunin and p23, a docking study was performed which revealed that the furan moiety of this compound forms hydrogen bonds with the side chain of Thr 90 and that Lys 95 hydrogen bonds with the epoxide of gedunin (docking score of 49) [27]. The anticancer effect of gedunin, as an Hsp90 inhibitor, will be discussed in detail for each type of cancer in its respective section.

#### 3.1.2. Gynecologic Cancers and Associated Cancers

Ovarian cancer is the fifth leading cause of death among women [49]. The first approach to the effect of gedunin on ovarian cell line was performed by Kamath et al. [28]. These authors studied the in vitro effect of gedunin on SKOV3, OVCAR4, and OVCAR8 ovarian cancer cell line proliferation, alone and in the presence of cisplatin. Their results revealed a noticeable decrease in cell proliferation after treatment with gedunin alone by more than 80% (*p* < 0.01), and with gedunin combined with cisplatin a decrease up to 47% compared with cisplatin treatment alone. In this study a bioinformatic analysis of integrated gedunin sensitivity and gene expression data was also carried out, with the discovery of 52 genes involved and related to modulation of cell survival and apoptosis pathways. The anti-proliferative potential of gedunin has also been investigated using the ID8, ID8TaxR, A2870, C30, and CP70 ovarian cancer cell lines [29]. In this study, the treatment with gedunin inhibited growth of all cell lines, and synergism between gedunin and paclitaxel was detected even at low concentrations (2.5 µM for each), which were not effective on cells when using each compound alone. Johnson et al. [29] were able to demonstrate that gedunin induced mitotic arrest between metaphase and anaphase, changing the expression of checkpoint kinase-1 (CHK1) and polo-like kinase-1 (PLK1) and resulting in apoptosis. It was also reported that gedunin-treated cells: (1) decreased inhibitory phosphorylation (Y15) of cyclin dependent kinase 1 (CDK1) and increased levels of cyclin B1, compared to untreated cells; (2) formed double-strand breaks; and (3) and increased the Bcl-2 to Bax protein ratio and mitochondrial cytochrome c release. 

Breast cancer is the most common cancer among women of reproductive age [49]. The anti-proliferative activity of gedunin against MCF-7 and SkBr3 breast cancer cells lines is reported by Brandt et al. [24]. In this study, gedunin, isolated from commercial neem oil, presented half maximal inhibitory concentration (IC_50_) values of 8.8 and 3.3 μM for MCF-7 and SkBr3, respectively. The effectiveness of gedunin, extracted from ground stems of *Cedrela odorata* L. grafted on *Toona cilata* var. *australis* (F. Muell.) Bahadur, against MCF-7 was also focused on in another study, where it presented a strong cell growth inhibitory activity against these cells (median growth inhibition concentration, GI_50_ = 9.1 μM) [31]. Two other studies evaluated the cytotoxic activity of gedunin, extracted from *A. indica* seed, against human breast cancer cell line SK-BR-3 and both found an IC_50_ value of 8.3 μM [32,33]. Patwardhan et al. [30] revealed that gedunin induced cancer cell death in cervical (HeLa-PR_B_) and breast carcinoma (MDA-MB-231, MDA-MB-453, Hs578T, T47D, and MCF-7) cell lines in a dose-dependent manner and selectively killed the cancer cells. These authors showed that the death pathway triggered by gedunin in MCF-7 and HeLa-PR_B_ cells was through caspase-7 activation and poly (ADP-ribose) polymerase (PARP) cleavage. By computational modeling, they revealed that gedunin can be successfully docked into the p23 structure, and that three amino acids (Thr-90, Ala-94, and Lys-95) can possibly mediate noncovalent interactions with the natural compound. Thus, Pathwardhan et al. [30] concluded that gedunin: (1) directly binds to p23 and inactivates it, without overexpression of Hsp27 and relatively modest induction of Hsp70; and (2) inhibits the p23 chaperoning activity, blocks its cellular interaction with Hsp90, and interferes with p23-mediated gene regulation. This leads to cancer cell death by apoptosis through inactivation of p23 and activation of caspase-7, which cleaves p23 at the C terminus.

These studies suggest that the treatment with gedunin, alone or combined with other chemotherapeutic drugs, can lead to promising results against gynecological and associated cancers. 

#### 3.1.3. Gastrointestinal Tract and Associated Cancers

Gedunin’s effect on oral cancer has been recently studied in vitro [35] and in vivo [34]. The in vivo approach investigated the effects of gedunin on the phosphoinositide 3-kinases (PI3K)/Akt/mammalian target of rapamycin (mTOR) and nuclear factor kappa B (NF-κB) signaling pathways, as well as hypoxia-inducible factor 1α (HIF1α)-mediated vascular endothelial growth factor (VEGF) signaling in a hamster buccal pouch (HBP) carcinogenesis model. For that, male Syrian hamsters with dimethylbenz(a)anthracene (DMBA)-induced oral cancer received intragastric administration of gedunin at different concentrations. It was observed that administration of gedunin, at 100 μg/kg bw), significantly (*p* < 0.01) reduced tumor incidence, and only mild hyperplasia was observed in the buccal pouches of the animals. Nagini and his co-workers [34] concluded that suppression of HBP was caused by inhibition of PI3K/Akt and NF-κB pathways through inactivation of Akt and inhibition of kappa B kinase (IKK), respectively, due to the inactivation of the aldose reductase (ARase) by gedunin. It blocked the angiogenesis by downregulating the expression of microRNA-21 (miR-21) and the pro-angiogenic factors vascular endothelial growth factor and HIF-1α. 

The in vitro approach used the SCC131 oral cancer cell line to investigate how gedunin, alone or combined with epalrestat, prevented the hallmarks of cancer by inhibiting ARase and the associated downstream PI3K/Akt/mTOR/ERK/NF-κB signaling axis. Tanagala et al. [35] concluded that gedunin, alone and combined, blocked cell proliferation, invasion, and angiogenesis, and induced cell death by blocking ARase-driven oncogenic signalling networks. Furthermore, gedunin combined with epalrestat was more effective than each single agent, suppressing the growth of SCC131 cells at half the concentrations used individually (0 to 180 μM for gedunin and 0 to 1.6 μM for epalrestat) [35].

Gedunin isolated from *A. indica* seed extract was evaluated for its cytotoxic activity against human stomach cancer cell line SK-BR-3 [33]. It presented moderate effectiveness against these cells, with an IC_50_ value of 16.9 μM. Moderate levels of cytotoxic activity were also observed on the CaCo-2 colon cancer cell line, tested with gedunin isolated from bark of *Xylocarpus granatum* J. Koenig, with an IC_50_ value of 16.8 μM [5]. Recently, Subramani et al. [36] studied the anti-proliferative effect of gedunin on pancreatic cancer cell lines (HPAC, PANC-1, and MIAPaCa-2), showing that it induced about 50% cell death at 25 μM in all of them. The same treatment revealed to induce apoptosis in all tested cancer cell lines with an increased expression of the pro-apoptotic markers Bax, as well as cleaved caspase-3, cleaved PARP, and cleaved caspase-8, and decreased in Bcl-2 expression. This means that gedunin induced both intrinsic and extrinsic mediated apoptotic cell death. In this study, Subramani et al. [36] showed that gedunin, at 15 μM, was able to inhibit the migratory abilities of all pancreatic cancer cells tested. The compound was also able to reduce the survival of pancreatic cancer cells by inhibiting the activation of PI3K and its downstream signaling via dephosphorylating PI3K at Tyr458/Tyr199, AKT at Ser473, p70S6K at Thr389, and mTOR at Ser2448. This study also showed that gedunin could inhibit three essential metastatic events, namely migration, invasion, and colony formation in pancreatic cancer cells, through epithelial-to-mesenchymal transition (EMT) inhibition due to the decrease in the expression of mesenchymal markers N-cadherin, Slug, Snail, vimentin, Notch 1 and 2, and Zeb increase in the expression of epithelial marker E-cadherin.

These different studies show that more research on gedunin as a new chemotherapeutic drug for gastrointestinal tract and associated cancers could be an interesting pathway to inhibit cancer cell proliferation, increase apoptosis, and, at the same time, inhibit metastatic characteristics of cancer cells, especially of the pancreas and mouth. 

#### 3.1.4. Prostate Cancer

The effect of gedunin on prostate cancer was assessed by Lamb et al. [37], who intended to elucidate the mechanism of action of gedunin as an AR inhibitor through a so-called Connectivity Map, using LNCaP cells. High connectivity scores were found for multiple instances of three Hsp90 inhibitors: (1) geldanamycin; (2) 17-allylamino-geldanamycin; and (3) 17-dimethylamino-geldanamycin, which had marked connectivity to the gedunin signature. Hsp90-interacting proteins were nearly entirely eliminated in gedunin-treated LNCaP, which means that gedunin was acting as an inhibitor of Hsp90 function, and was thus hypothetically inhibiting AR expression (as its stability is dependent on Hsp90 activity). Later, using a similar approach and the LNCaP cell line as well, it was shown that gedunin inhibited Hsp90 activity and Hsp90 clients, including AR [38]. Boopalan et al. [39] demonstrated that gedunin inhibited the growth of cancer cells and decreased the expression of Hsp90 and phosphorylation of mTOR and 4EBP. Gedunin also induced autophagy and apoptosis simultaneously by regulating key factors like LC3B, WIPI-1, VMP-1, pJNK, cleavage of caspases, and PARP.

#### 3.1.5. Lung Cancer

Gedunin extracted from ground stems of *C. odorata* showed a strong cell growth inhibitory activity against non-small cell lung cancer NCI-H460, with a GI_50_ value of 8.36 μM [31]. In this study, the underlying mechanism of the growth inhibitory activity of gedunin was also investigated for its effects on cell cycle profile and NCI-H460 programmed cell death. Cazal et al. [31] concluded that gedunin arrested the cell cycle progression in the S phase, leading to a decrease in the percentage of cells in the G1 phase, thus inducing apoptosis. Recently, the anti-proliferative activity of free and nanoencapsulated gedunin against human non-small-cell lung cancer (NCI-H292) cells was investigated by Nwokwu et al. [41], demonstrating that liposomal gedunin has greater anti-proliferative effects in NCI-H292 cells than free gedunin, with IC_50_ values ranging from 3.4 to 1.8 μg/mL (24 to 72 h) and 26.4 to 21.6 μg/mL (24 to 72 h), respectively. These authors also studied the apoptotic effect of gedunin-loaded liposomal nanoparticles in NCI-H292 cell line and concluded that it enhances anti-proliferative effects through *p53*-initiated, *Bax*-associated, caspase-dependent activation of apoptosis. Another recent study, by Hasan et al. [40], demonstrated that gedunin exerts cytotoxic effects on A549 cells in a dose-dependent manner after 24 h of treatment, and that this cytotoxic potential was in agreement with hallmarks of apoptosis, as gedunin increased reactive oxygen species (ROS) generation by 8.2 fold, loss in mitochondrial membrane potential by 3.6-fold, and increased chromatin condensation by 5.3 fold, as compared to the untreated control. These authors concluded that gedunin, as an inhibitor of Hsp90, regulates the PI3K/AKT signaling pathway, inhibits autophagy, and induces apoptosis in lung cancer cells.

These different studies show that gedunin, encapsulated or not, should be taken into consideration as a new possible therapeutic agent of non-small-cell lung carcinoma.

#### 3.1.6. Brain Cancer

Glioma is the most frequently detected primary brain tumor found in adults [50]. Recently, Li et al. [42] studied the effect of gedunin on viability, migration, and invasion of the U-251 MG cell line. Gedunin, at 20 μM, reduced the viability of the cells to 28% and significantly (*p* < 0.002) inhibited their invasive and migratory tendency. It also caused inhibition of MMP-9, Rho-associated protein kinase 1 (ROCK-1), and focal adhesion kinase (FAK) mRNA expression, as well as significant reduction of uPA protein expression. Therefore, gedunin can be considered as a possible new agent for the treatment of glioma.

#### 3.1.7. Leukemia

Cytotoxic activity of gedunin, from *A. indica* seed extract, was evaluated against human leukemia cell line HL60, and an IC_50_ value of 5.9 μM was observed [32,33]. Sakamoto et al. [43] using gedunin extracted from *Carapa guianensis* Aubl. flower oil, assessed its cytotoxicity against murine P388 leukaemia and HL60 cell lines, and obtained IC_50_ values of 16 and 15.2 mM, respectively.

#### 3.1.8. Skin Cancer

The B16 murine melanoma cell culture model was used to investigate cytotoxic effect of gedunin extract from the seeds of *A. indica*. Gedunin treatment (25 μg/mL) resulted in moderate melanogenesis inhibitory activity and cytotoxicity, a 93% reduction of melanin content, and 13% cell viability, respectively [44]. A similar study revealed that gedunin treatment (10 μg/mL) resulted in a 72% reduction of melanin content and 85% cell viability, with an activity-to-cytotoxicity ratio of 0.34 [32], thus presenting a melanogenesis-inhibitory activity. Gedunin extracted from ground stems of *C. odorata* showed a strong cell growth inhibitory activity against melanoma cell line A375-C5, with a GI_50_ value of 8.8 μM [31].

#### 3.1.9. Cancer Stem Cells

Cancer stem cells (CSCs) are considered as initiators of tumor development and progression, as they can renew themselves indefinitely, generate new tumors, and cause metastasis and relapse [51]. An in silico molecular docking simulation with gedunin was carried out for its binding with receptor proteins involved in the main signaling pathways of CSCs and revealed a drug likeness of gedunin with β-catenin chain A in cancer stem cells [52]. The in vitro effectiveness of gedunin on human embryonal carcinoma (NTERA-2) cells, as a cancer stem cell model, was later evaluated, indicating a dose- and time-dependent inhibition of NTERA-2 cell proliferation treated with gedunin [45]. The compound also exerted a potential anti-proliferative effect on these cells, with IC_50_ values of 14.59, 8.49, and 6.55 μg/mL at 24, 48, and 72 h after incubations, respectively. Gedunin inhibited the expression of Hsp90, its client proteins, and *survivin*, and upregulated *Bax* and *p53*. The apoptotic effect of gedunin was confirmed by DNA fragmentation, with increased caspase-3 and caspase-7 activity and morphological changes related to apoptosis. These results revealed that gedunin may be a good candidate for the development of CSC-targeted anti-cancer drugs.

### 3.2. Neuroprotective Activity

Neuroprotection is the relative protection of neural structure and/or function. Many central nervous system (CNS) disorders have common mechanisms, such as oxidative stress, excitotoxicity, inflammatory changes, and mitochondrial dysfunction [53].

The Hsp90 complex has long been associated with neuropathological phenotypes related to Parkinson’s disease (PD) and its inhibition in neuroprotective in disease models. Prolyl hydroxylase domain protein 2 (PHD2) is the primary regulator of steady-state levels of the transcription factor HIF1α. PHD2 levels have been reported to be elevated within affected substantia nigra pars compacta (SNpc) tissues isolated from PD patients in conjunction with reductions in HIF1α levels [54]. The Hsp90 co-chaperone p23 is able to recruit and stabilize PHD2, thus reducing HIF1α neuroprotective effect levels and its targets. Recently, the p23 inhibitor gedunin was found to protect against neurotoxicity associated with 1-methyl-4-phenylpyridine (MPP^+^) in dopaminergic (DAergic) N27 cells [55]. Gedunin binds to p23 and prevents its interaction with Hsp90, thus inhibiting Hsp90 chaperone activity, without interfering with any other known Hsp90 co-chaperons. Gedunin could provide a new alternative in the treatment of PD, which may have fewer broad effects than other Hsp90 inhibitors.

Alzheimer’s disease (AD) is one of the most common neurodegenerative disorders worldwide. Histopathological hallmarks of AD include extracellular amyloid plaques and intracellular neurofibrillary tangles (NFTs). Their accumulation leads to neurodegeneration in the hippocampus and cortex, and activation of microglia cells. In progressive brain damage, these cells can become chronically activated, resulting in a sustained aberrant inflammatory response [56]. Taking this into consideration, and knowing that gedunin has been: (1) proven to have neuroprotective effects against the toll-like receptor (TLR)-mediated inflammation (TLR2, TLR3, and TLR4) through the regulation of proinflammatory inflammasome activation and cytokine production [57,58]; and (2) has also been reported as a nuclear factor erythroid 2-related factor 2 (Nrf2) activator, inducing astrocyte-dependent neuroprotection from oxidative stress via a Nrf2-dependent mechanism [59], Tom et al. [56] studied the effect of gedunin on oligomeric Aβ_1–42_-induced microglial activation and subsequent inflammation. From this study, three main conclusions can be highlighted: (1) gedunin suppressed, in a microglial cell line, neuroinflammation arising from Aβ_1–42_ oligomer exposure, inducing NF-κB activation and its targets, including nitric oxide (NO) and IL-1β (interleukin 1, beta); (2) gedunin inhibited neuroinflammation by activating Nrf2 and its downstream targets γ-glutamylcysteine synthetase, heme oxygenase 1, and NADPH quinone dehydrogenase 1, involved in quenching reactive oxygen and nitrogen species generated by NF-κB activation; and (3) gedunin prevented, in human neuronal cells (SH-SY5Y), neurotoxicity secondary to Aβ-induced microglial activation. Hereupon, gedunin can be seen as a natural alternative therapy against neurodegenerative diseases such as AD.

### 3.3. Antidiabetic Activity

Diabetes mellitus (DM) is a metabolic disorder caused by a defect in the insulin secretion and/or action, leading to chronic hyperglycemia [60]. Human pancreatic α-amylase (HPA) is a key enzyme in the digestive system and plays an important role in DM [61]. It catalyzes the initial step in the hydrolysis of starch, which is a principal source of glucose in the diet. Thus, HPA inhibitors present an effective strategy to lower postprandial hyperglycemia by controlling starch breakdown [61]. Recently, neem limonoids, including gedunin, were screened and identified as potent inhibitors of HPA, based on their inhibition potency and in vitro cytotoxicity, and studied in order to identified their mode of action and underlying molecular interactions [62]. In this study, gedunin presented promising results as an HPA inhibitor. Gedunin exhibited a porcine pancreatic α-amylase inhibition IC_50_ value of 72.2 μM, cytotoxicity at an IC_50_ value of 13.4 μM for AR42J cell line, and amylase inhibition of 69.2%, with maximal secreted α-amylase inhibition of 53.4% seen at 3.3 μM. In silico screening of gedunin docked onto HPA revealed a minimal energy of interaction of −25.9 kJ/mol and the involvement of a pi-alkyl interaction between Trp 58 and the C29 methyl of the A ring, with a bond length of 5.30 Å. This suggests that any substitution in the A ring may interfere in gedunin binding to HPA. In this study, gedunin inhibited HPA with sigmoidal fit with an IC_50_ value of 68.38 μM, and the Michaelis–Menten kinetics suggested a mixed mode of inhibition with maltopentose (K*i* 18.6 μM) and starch (K*i* 37.4 μM) as a substrate with a stoichiometry of 1:1. Results also suggested that gedunin likely binds near the active pocket. However, the active site residues were not involved in their binding. Fluorescence and circular dichroism spectroscopy confirmed the involvement of tryptophan and tyrosine in ligand binding to HPA. The interaction between HPA and gedunin was a spontaneous process with free energy decreasing; the binding was enthalpically and entropically driven with ΔG° of −21.16 kJ/mol. Thus, gedunin can be seen as a HPA inhibitor molecule and it can aid in the design of better drug candidates with newer inhibitors of HPA for controlling starch digestion in order to reduce post-prandial hyperglycemia.

### 3.4. Antiallergic Activity

Allergy is characterized by a strong inflammatory response involving several mediators and cell types [63]. Oil extracted from *C. guianensis* seeds, containing gedunin, among other effects, inhibited edema formation during the allergic response in vivo [64]. This property was shown to be mediated by the inhibition of major inflammatory mediators involved in vascular permeability, such as histamine, platelet activating factor (PAF) and bradykinin. This oil also inhibited allergic eosinophilia, which is correlated with the inhibition of CCL11/eotaxin and IL-5 generation through the NFκB signaling pathway impairment in mice [65]. Ferraris et al. [63] revealed that gedunin has an important antiallergic activity in in vivo models of allergic airway inflammation, achieved by both pre- and post-treatments with gedunin. This effect showed to be mediated by the inhibition of chemotactic mediators involved in T cell and eosinophil migration. Gedunin directly modulated T lymphocyte activation and trafficked into the airways, which is critical for the development and maintenance of the inflammatory allergic response. Regarding these studies, it can be said that gedunin might represent a potential therapeutic tool to suppress allergic features and control allergic diseases.

### 3.5. Insecticidal, Herbicidal, Antifeedant, and Nematicidal Activity

Natural pesticides are biodegradable and eco-friendly, as some are described as having less environmental impact than most commercial chemical insecticides [66]. Gedunin’s properties as a biopesticide have long been tested against *Spodoptera frugiperda* and it proved to cause significant larval mortality, as well as growth reduction, with a half maximal effective concentration (EC_50_) ranging from 10 to 39 ppm [14,67,68,69,70,71]. Gedunin’s antifeedant activity against *Cnaphalocrocis medinalis* was widely studied. Gedunin not only affects *C. medinalis* growth and larval behavior, but also affects its gut enzyme activity, such as acid phosphatases (ACP), alkaline phosphatases (ALP), adenosine triphosphatases (ATPase), and lactate dehydrogenase (LDH) [66,72,73,74]. The half maximal lethal concentration (LC_50_) value obtained for gedunin against the fourth-instar larvae of *Anopheles stephensi* was 120 ppm [75]. For the same agent, the larvicidal, pupicidal, adulticidal, and antiovipositional activity of gedunin was studied, and it presented biological activity at high doses and the EC_50_ against first to fourth instar larvae of *A. stephensi* ranged from 0.058 to 0.117 ppm [76]. Gedunin has also been tested against *Ostrinia nubilalis*, presenting antifeedant activity at 10–50 ppm [77,78]. *Reticulitermes speratus* presented growth inhibition in the presence of gedunin (PC_95_ = 218.4 μg/disc) and of a mixture of limonoids containing gedunin (PC_95_ = 0.35% *w/w*), isolated from a neem oil extract [79,80]. For *Helicoverpa armigera*, gedunin presented EC_50_ between 3.51 and 50.8 ppm and for *Spodoptera litura* an EC_50_ of 40.4 ppm [81,82]. Gedunin was also proven active against *Sitophilus oryzae* at 0.50% (*w/w*) [83], *Pectinophora gossypiella,* and *Heliothis zea*, with an EC_50_ of 32 and 50 ppm, respectively [67].

More recently, gedunin’s toxic action against instar larvae of *Aedes aegypti*, the mosquito responsible for transmission of yellow fever and dengue fever, and of *Culex quinquefasciatus*, the mosquito responsible for filariasis transmission, was determined. Gedunin, isolated from neem seed kernel oil, exhibited 100% toxic action against both mosquito larvae at 50 and 100 ppm [84]. The pesticidal activity of gedunin was tested in four different insects, *Nilaparvata lugens*, *Myzus persicae*, *S. litura,* and *Plutella xylostella* [85]. It showed insecticidal activity to *N. lugens* with 40, 20, and 0% mortality at concentrations of 1, 0.5, and 0.25 mg/mL, respectively, and moderate activity against *P. xylostella*, with 60, 40, and 10% mortality at concentrations of 1, 0.5, and 0.25 mg/mL, respectively. Gedunin did not show insecticidal or larvicidal activities against *S. litura* and *M. persicae*. Due to its recognized power as a natural insect growth inhibitor, gedunin has also been commonly used as a positive control in several antifeedant activity studies against *S. frugiperda*, *Drosophila melanogaster*, *Acanthoscelides obtectus*, and *Epilachna varivestis* [86,87,88,89].

Other bioactivities have been tested on gedunin, namely nematicidal and herbicidal activities. In the first case, gedunin was tested with *Meloidogyne incognita* and presented 40% mortality at 100 ppm [90]. In the second case, etiolated wheat coleoptile bioassay, which detects the effects on development of non-differentiated vegetal cells, was used to evaluate phytotoxicity of gedunin, isolated from the stem of *C. odorata* grafted onto *T. ciliata* var. *australis* [91]. The compound presented a good inhibition effect for all tested concentrations, with an IC_50_ of 2.642 μM, and thus was selected for phytotoxicity evaluation on the standard target species (STS) *Lepidium sativum* L. (cress), *Lactuca sativa* L. (lettuce), *Lycopersicon esculentum* Mill. (tomato), and *Allium cepa* L. (onion). Gedunin affected the root growth of *L. sativum*, at all tested concentrations, with similar levels to that of the herbicide Logran^®^ (used as positive control). However, its behavior was not significant for the other STS.

Gedunin extracted from different plants of the Meliaceae family, neem (seeds, oil) and *Cedrela* species (wood, stem) proved to be effective at eradicating several larvae. This way, gedunin can be considered an eco-friendly pest management tool.

### 3.6. Antibacterial and Antifungal Activity

Antifungal activity of gedunin was first tested against *Polyporus palustris* and *Polyporus versicolor* [92]. Gedunin, isolated from *Xylocarpus obovatus* A. Juss., showed a marked and progressive antifungal activity, with *P. palustris* being more sensitive than *P. versicolor*. More recently, fungicidal activity of gedunin was determined against six phytopathogenic fungi: *Pyricularia grisea*, *Rhizoctonia solani*, *Botrytis cinerea*, *Phytophthora infestans*, *Puccinia recondite*, and *Erysiphe graminis* [85]. In this study, the whole plant method was used for three different gedunin concentrations: 1, 0.5, and 0.25 mg/mL. Gedunin only exhibited antifungal activities of 40 and 20% to *P. recondite*, at concentrations of 1 and 0.5 mg/mL, respectively. Antibacterial activity of gedunin was determined for a Gram-negative bacterium, *Xylella fastidiosa*, which presented a minimum inhibitory concentration (MIC) value of 2.7 × 10^3^ μM [93].

Gedunin has been proved to have antifungal and antibacterial activity. Although, more tests could be performed and gedunin could also be tested in combination with antibiotics.

### 3.7. Antiparasital Activity

The first study on gedunin’s antimalarial activity was published in 1986 [94]. The antimalarial screening was performed against *Plasmodium falciparum*, the major human malaria parasite. Gedunin isolated from *A. indica* had an IC_50_ value of about 1 μM after 48 h exposure (0.3 μM after 96 h), equivalent to quinine. Gedunin presented an IC_50_ of 0.72 μg/mL in an in vitro assay against chloroquine resistant K1 strain of *P. falciparum*, but in an in vivo assay neither oral nor subcutaneous administration of this compound to mice infected with *P. berghei*, in a 4-day test at the dose of 90 mg/kg/day, resulted in inhibition of parasitemia [95]. Against two clones of *P. falciparum*, one sensitive to chloroquine (D6) and one chloroquine-resistant (W2), gedunin (isolated from *C. odorata* wood) presented an IC_50_ of 39 and 20 ng/mL, respectively, having better activity against the resistant clone than chloroquine or quinine [22]. Gedunin isolated from bark of *Khaya grandifoliola* C. DC. proved to be effective against *P. falciparum* (W2/Indochina clone), with an IC_50_ of 1.25 μg/mL, and also to some *P. falciparum* clinical isolates from symptomatic Cameroonian patients, with IC_50_ values ranging from 3.36 to 8.89 μg/mL [96]. Drug interaction between chloroquine and gedunin was also studied against *P. falciparum* (W2/Indochina clone) and gedunin exhibited an additive effect. Lee et al. [97] also tested gedunin against W2-strain using a [^3^H]-hypoxanthine and 48-h culture assay in vitro; it presented antimalarial activity with IC_50_ values of 3.1 and 0.14 μM, respectively. Gedunin isolated from *A. indica* and *X. granatum* fruits showed in vitro antimalarial activity. In the first case, IC_50_ values of 1.66 and 1.31 μM were observed for the *P. falciparum* D10 strain and W2 strain, respectively [98]. In the second case, the MIC of gedunin was determined for the *P. falciparum* 3D7 strain, at 10 μg/mL [99]. A limonoid-rich fraction from *C. guianensis*, containing gedunin, showed to be effective against W2 and Dd_2_ strains of *P. falciparum* [100]. The fraction inhibited growth of the W2 clone in 100% at concentration of 3.1 μg/mL (24 and 48 h), and of the Dd_2_ clone in 56 and 82% (24 and 48 h), with an IC_50_ value of 2.8 μg/mL and 0.4 μg/mL, respectively.

In vitro antimalarial activity of gedunin has been widely studied and proved. However, in vivo, gedunin, micronized in water and administered to mice infected with *P. berghei*, proved not to be as effective [95]. This may be explained by the (1) poor solubility of gedunin, due to its lipophilicity; (2) first pass metabolism of gedunin by cytochrome P450 enzymes of the small intestine, which reduces plasma levels of drugs; and (3) hydrolysis of gedunin by the inactive and unstable metabolite, 7-deacetylgedunin [22]. In order to improve in vivo gedunin antimalarial activity, Omar et al. [23] used a combination of gedunin with dillapiol, a cytochrome P450 inhibitor, and also tested a structural modification of the compound. They concluded that gedunin was slightly active in parasite-infected mice due to poor pharmacokinetics. A synergistic treatment of 50 mg/kg/day gedunin with 25 mg kg/day/dillapiol increased parasitemia clearance in mice to 75%, with the addition of a stable methoxy group at the C-7 position and a 67.5% suppression rate.

Gedunin was revealed to be one of the most potent antimalarial limonoid. The conjugated enone system, the furan ring, and the acetoxy group at C-7 have been identified as critical for the antimalarial activity of gedunin, as well as the Michael acceptor in ring A [22]. More studies on the mechanisms of gedunin’s antimalarial action, which remains elusive, could allow a better understanding of the structural requirements needed by an active molecule. Recently, an in silico study determined the binding affinities of limonoids, including gedunin, to the protein kinase 5 of *P. falciparum* (PfPK5) [101]. This protein kinase is described as necessary for the activation and maintenance of S-phase of the parasite, thus being recognized as one of the novel targets in *falciparum* species. All the molecules tested were able to bind to PfPK5 and gedunin was the second best of the test, with a binding-energy of −5 kcal/mol, right after 7-deacetoxy-7-oxogedunin and nimolicinol (−5.5 kcal/mol, each). This was comparable to the result obtained for staurosporine, a known protein kinase inhibitor, at −4.66 kcal/mol. This result means that gedunin was able to fit into the binding cavity of the targeted protein and interact with the protein molecule via a hydrogen and pi-bond [101]. This study suggested that gedunin, along with other limonoids, could be a potential pfpk5 inhibitor, which could further be developed into antimalarial drugs.

Gedunin has also shown activity against human lymphatic filarial parasite, *Brugia malayi*. The compound was isolated from *X. granatum* fruits and tested in vitro and in vivo against the mentioned parasite [102]. Gedunin presented a good activity in vitro on both adult parasite and microfilaria, with IC_50_ values of 0.24 and 2.03 μg/mL, respectively. In vivo, gedunin caused 80% mortality of adult worms in a 5-day test at 100 mg/kg/day.

The activity of gedunin on *Babesia* and *Theileria* parasites was evaluated, for the first time, using an in vitro model by Azirwna et al. [103]. These authors used three strains of *Babesia* and one of *Theileria*, namely *B. bovis* (Texas strain), *B. bigemina* (Argentina strain), *B. caballi* (USDA strain), and *T. equi* (USDA strain). Gedunin inhibited the growth of *Babesia* parasites and the IC_50_ values were moderately low: 21.7, 15.3, and 22.1 μM for *B. bovis*, *B. bigemina,* and *B. caballi*, respectively; and 33.2 μM for *T. equi*. Morphological and degenerative changes were also observed in parasites treated with gedunin. An alternative method was later developed and optimized to microscopy screening of *Babesia*, a fluorescence-based method [104,105]. The newly developed method for drug evaluation against *B. bovis* was successfully achieved; the IC_50_ value obtained, 19 μM [104], was comparable to the previously published microscopy-based value, 21.7 μM [103], with no statistically significant (*p* < 0.05) difference between them. Initially, this method was not suitable for a large scale screening, since medium had to be replaced every day, but that was later overcome [105].

### 3.8. Other Activities

The effect of gedunin, isolated from powdered fruits of *X. granatum*, on H^+^ K^+^-ATPase (proton pump) inhibitory activity in isolated gastric microsomes from rat stomach was studied by Lakshmi et al. [106]. Gedunin showed gastroprotective effects through the inhibition of proton pump activity with an IC_50_ of 56.86 μg/mL. Thus, more studies could be performed as it can be considered a possible therapeutic agent in treating gastric ulcer.

According to Hullin-Matsuda et al. [107], gedunin inhibited more than half of de novo synthesis of sphingomyelin biosynthesis and ceramide transport protein (CERT)-mediated extraction of ceramide from endoplasmatic reticulum membrane. Ceramide has a central position in sphingolipid metabolism, which has been associated with cancer cell proliferation and *Plasmodium* development. Thus, gedunin’s inhibition of sphingomyelin biosynthesis also represents a reasonable explanation for its anticancer and antimalaria properties. The fact that gedunin stimulates suicidal erythrocyte death or eryptosis, is also useful for anticancer and antimalaria therapy. This was proved by the treatment of human erythrocytes with gedunin, for 48 h, which revealed that it significantly increased cytosolic Ca^2+^ activity and annexin-V-binding, and significantly decreased cell volume [108].

Known as an Hsp90 inhibitor, gedunin was used in a study related to the Duchenne and Becker muscular dystrophies [109]. The inhibition of Hsp90 and, consequently, the release of bound HSF1 for transcriptional activity, increased L54R GFP-dystrophin in cells treated with 10–80 μM gedunin. This suggests that pharmacologic activation of the heat shock system can facilitate stabilization of mutant dystrophin. The effectiveness of gedunin supports the idea of heat shock activators as a class of compounds with the potential to treat Duchenne muscular dystrophy patients with missense mutations.

A pool of six different tetranortriterpenoids containing gedunin, extracted from *C. guianensis* seed oil, was demonstrated to have anti-inflammatory activity, including articular inflammation, by inhibiting zymozan-induced knee joint inflammation [110]. More recently, Conte et al. [111] demonstrated that in vivo pre- and post-treatment with gedunin impaired several features of murine zymosan-induced arthritis in mice, including knee joint swelling, neutrophil influx, hyperalgesia, and production of inflammatory mediators. The compound directly damages neutrophil and macrophage activation by impairing calcium influx, chemotaxis, cell adhesion, and lipid body formation. Gedunin, isolated from flower oil of *C. guianensis*, presented anti-inflammatory activity by strongly affecting the production of NO in lipopolysaccharide (LPS)-activated mouse peritoneal macrophage (IC_50_ value of 4.6 μM) [112] and had a protective effect against liver injury induced by d-galactosamine (d-GalN)/LPS in mice at a dose of 25 mg/kg, p.o [113]. Ninomiya et al. [113] believe that the mechanism of action of gedunin may involve inhibition of LPS-induced macrophage activation and reduction of sensitivity of hepatocytes to tumor necrosis factor-α (TNF-α), despite the fact that gedunin did not decrease the cytotoxicity caused by d-GalN. Recently, Morikawa et al. [114], found that gedunin, isolated from *C. guianensis* seed oil, significantly promoted collagen synthesis in normal human dermal fibroblasts to 133.3, 134.7, and 136.8% at 3, 10, and 30 μM, respectively, without cytotoxicity at the same concentrations [114].

## 4. Toxicity

Natural products from plants of Meliaceae family, especially from *A. indica*, have been safely consumed for several years [49]. However, it is important to know if this safety is extended to one of the pure compounds that can be extracted from those plants, gedunin. Its toxicity is not well characterized in either in vitro or in vivo models.

Gedunin was relatively non-toxic to guinea-pig ear epidermis (GPK) cells, with a median effective dose (ED_50_) of 275.10 μg/mL, and no toxicity was observed during its oral or subcutaneous administration at 90 mg/kg/day to mice, in a 4-day test [95]. Omar et al. [23] studied the pharmacokinetics of gedunin in order to determine its absorption, distribution and excretion in a mammalian model, using 12 male Sprague-Dawley rats (230–240 g) and administered radiolabeled gedunin at a dose of 50 mg/kg orally by gavage, providing a radioactive dose of approximately 6 × 10^6^ dpm per kg body weight. Blood was collected from the tail vein at 1, 2, 3, 6, 9, 12, 24, 48, 72, and 96 h and serum-analyzed for gedunin content. Poor absorption of the orally administered gedunin and rapid clearance of label from the blood were observed. The acute toxicity of gedunin was also investigated using five CD-1 male mice (30 ± 2 g), each receiving a daily dose of 100 mg/kg of gedunin and 25 mg/kg of dillapiol for 5 days. Weight changes were recorded every day for 14 days and, on the last day, blood was collected via cardiac puncture. Results showed no significant differences (*p* < 0.05) in total body or organ weight, gross anatomy or three liver enzyme levels relative to the control group, thus suggesting that the treatment had no acute mammalian toxicity.

Patwardhan et al. [30] revealed that adult-derived normal cells, Hs578Bst cells, and human mammary epithelial cells (HMEs) were remarkably resistant to gedunin treatment and that by comparing cell growth of cancer versus immortalized normal cell lines, gedunin selectively killed cancer cells. A similar result was obtained for pancreatic cells. Subramani et al. [36] showed that 25 μM of gedunin were not able to significantly alter cell viability of normal pancreatic cells (hTERT-HPNE), and microscopic analysis also showed minimal or no reduction in cell number of hTERT-PNE treated. However, the same concentration of gedunin was able to induce approximately 50% cell death in three pancreatic cancer cell lines, suggesting that gedunin selectively kills pancreatic cancer cells and is nontoxic to normal ones.

The effects of gedunin on normal human cells were evaluated by WST-1 cell viability assay on human peripheral blood mononuclear cells [45]. The IC_50_ value obtained was >100 μg/mL for all incubation periods tested (24, 48, and 72 h), which means gedunin is not cytotoxic to these cells. Nwokwu et al. [41] tested gedunin and gedunin-loaded liposomal nano-particles on normal human lung fibroblast (MRC-5) cells at different experimental timelines 24, 48, and 72 h. The compound itself presented IC_50_ values of > 50 μg/mL at all times tested and gedunin liposomes presented IC_50_ values of 5.32, 2.94, and 4.90 μg/mL for 24, 48, and 72 h, respectively. Nevertheless, gedunin liposomes were less toxic than the control drug used, paclitaxel (*p* < 0.05).

An in silico investigation evaluated gedunin’s pharmacokinetics and toxicity [101]. The pharmacokinetic parameters and toxicity of selected biomolecules were predicted using admetSAR in different model and an online server, ABSYNTH, was used to predict *Escherichia coli* toxicity. The latter helps to predict the concentration of drug molecule required to inhibit the growth of the prokaryotic cell, and the value obtained for gedunin was 0.37 g/L. In terms of pharmacokinetic parameters, this study showed that gedunin had a positive result to cross blood–brain barrier and for human intestinal absorption, with a probability of 0.85 and 1, respectively. Regarding Caco-2 permeability, Ames toxicity, and carcinogenicity, gedunin presented negative results to all with a probability of 0.56, 0.85, and 0.92, respectively. For acute oral toxicity gedunin was under class III with a probability of 0.37. According to these pharmacokinetic results, gedunin is said to be suitable for human consumption. This study also predicted a median lethal dose (LD_50_) of 3.07 mol/kg and a pIGC_50_ of 1.27 μg/L for rat and *Tetrahymena pyriformis*, respectively.

## 5. Materials and Methods

A literature search using several online databases, such as PubMed, Science Direct, ISI Web of Knowledge, and Google scholar, was conducted up to September 2019. The principal search topics were related to gedunin and Meliaceae limonoids: its isolation, biological activities, toxicity, and chemistry. Secondary searches included articles cited in sources identified by the previous search.

## 6. Conclusions and Future Perspectives

From the studies presented and highlighted in this review, it is clear that gedunin, an important limonoid from the Meliaceae family, represents a natural compound with a great potential in terms of bioactivities, some of them of great value to prevent and treat a wide range of human diseases. The first studies on gedunin’s biological activities were focused on antifungal, antibacterial, antiparasital and insecticidal, herbicidal, antifeedant, and nematicidal capacities. The discovery of gedunin as an Hsp 90 inhibitor represents a very important milestone for its study as a biological therapeutic agent. This finding opened the door to another therapeutic side of gedunin, especially its anticancer and neuroprotective aspects.

There few studies about gedunin’s antibacterial and antifungal activity, and it proved to be an effective agent. Newer studies against other pathogenic agents are required, for example, those on the WHO priority pathogens list. These studies should focus not only on the determination of gedunin’s activity against microbiological agents, but also on its mechanism of action. Gedunin has also been proven to be a bioactive compound with respect to crop protection, and is effective against several pests. This represents an important future area of research that should focus on documenting the role of gedunin as an alternative to conventional chemical pesticides and insecticides, demonstrating its efficacy under field conditions, toxicity to mammalian, persistence in the environment, and biodegradability.

Gedunin has the potential to prevent or treat several human cancers, and its cytotoxic activity can be explained by multiple cellular and molecular mechanisms, including cell proliferation inhibition, apoptosis induction, and effect on tumor invasion and angiogenesis. A careful examination of the in vitro and in vivo anticancer studies presented here reveals that gedunin is effective in preventing or treating oral, stomach, colon, pancreatic, breast, ovarian, prostate, lung, brain, skin, leukemia, and stem cell cancers. However, further studies should be done in order to better understand the specific mechanism to treat each type of cancer. Another area of interest in this research field concerns the use of advanced technology such as liposomal drug delivery and nanoformulation to improve the efficacy of gedunin in cancer treatment, as well as the use of gedunin combined with other chemotherapeutic drugs. Other human diseases such as AD, PD, DM, and malaria have found a new pathway of treatment through gedunin. In these cases, like for anticancer and also anti-inflammatory activity, further studies need to be conducted in order to better understand the specific mechanism leading to the treatment, and to also prove gedunin’s safety for human consumption by performing pre-clinical and clinical trials.

In conclusion, gedunin shows significant promise for preventing and treating several human diseases, thus having a high commercial importance as well, but more studies need to be performed on its efficacy. Another important issue to note is that compounds like gedunin are difficult to produce at a quantity high enough from nature, and cannot be easily supported by chemical synthesis, which represents a great opportunity for commercial laboratories and for researchers.

## Figures and Tables

**Table 1:** Comparative anticarcinogenic properties of gedunin in different types of cancer.

Cancer Type		Gedunin Source	Type of Study		Effect	Mechanisms	References
			In Vitro	In Vivo			
Gynecological cancers and associated cancers	Ovarian cancer	Commercial	SKOV3 OVCAR4 OVCAR8	-	Inhibits cell proliferation	An up to 80% decrease in cell proliferation; bioinformatic analysis identified 32 genes involved	[28]
			ID8 ID8TaxR A2870 C30 CP70	-	Inhibits cell proliferation Apoptosis induction	Induces mitotic arrest between metaphase and anaphase; changes the expression of checkpoint kinase 1 (CHK1) and polo-like kinase-1 (PLK1); decreases inhibitory phosphorylation (Y15) of cyclin dependent kinase 1 (CDK1) and increases levels of cyclin B1; induces DNA fragmentation and increases the Bcl-2 to B-cell lymphoma-2 protein associated X protein (Bax) protein ratio and mitochondrial cytochrome c release	[29]
	Breast and cervical cancer	Commercial neem oil extract	MCF-7 SK-BR-3	-	Inhibits cell proliferation	Inhibits Hsp90	[24]
		Commercial	HeLa-PR_B_ Hs578T MCF-7 MDA-MB-231 MDA-MB-453	Mouse embryonic fibroblast culture	Apoptosis induction	Mediates caspase-7 cleavage of the co-chaperon p23	[30]
		Ground stem extract (*Cedrela odorata*)	MCF-7	-	Exhibits cytotoxic effects	-	[31]
		Seed extract (*Azadirachta indica*)	SK-BR-3	-	Exhibits cytotoxic effects	-	[32,33]
Gastrointestinal tract and associated cancers	Oral cancer	Commercial	-	Male Syrian hamsters with 7,12-dimethylbenz(a)anthracene (DMBA)-induced oral cancer	Effects on tumor invasion and angiogenesis	Inhibits kinases Akt, inhibitory kappa B kinase (IKK), and aldose reductase (ARase), and the oncogenic transcription factors nuclear factor kappa B (NF-κB) and hypoxia inducible factor 1 alpha (HIF1α)	[34]
			SCC131	-	Inhibits cell proliferation Apoptosis induction	Blocks cell proliferation, invasion, and angiogenesis, and induces cell death by blocking the ARase-driven oncogenic signaling network	[35]
	Stomach cancer	Seed extract (*A. indica*)	AZ521	-	Exhibits cytotoxic effects	-	[33]
	Colon cancer	Bark extract (*Xylocarpus granatum*)	CaCo-2	-	Exhibits cytotoxic effects	-	[5]
	Pancreatic cancer	Commercial	HPAC MIAPaCa-2 PANC-1	Female athymic nude mice with HPAC-induced carcinogenesis	Inhibits cell proliferation Apoptosis induction Effects on tumor invasion and angiogenesis	Induces both intrinsic and extrinsic mediated apoptotic cell death; inhibits the activation of phosphoinositide 3-kinases (PI3K) and its downstream signaling by dephosphorylating PI3K at Tyr458/Tyr199, Serine/threonine kinase (AKT) at Ser473, p70S6K at Thr389, and mammalian target of rapamycin (mTOR) at Ser2448; inhibits metastasis of pancreatic cancer cells by decreasing their epithelial-to-mesenchymal transition (EMT) as well as invasive, migratory, and colony formation capabilities, and also inhibits sonic hedgehog signaling pathways	[36]
Prostate cancer		Commercial	LNCaP	-	Inhibits cell proliferation	Inhibits heat shock protein 90 (Hsp90) activity	[37]
						Inhibits Hsp90 activity and Hsp90 clients, including androgen receptor	[38]
		Not specified	Not specified	-	Inhibits cell proliferation Apoptosis induction	Decreases expression of Hsp90 and phosphorylation of mTOR, 4EBP; regulates LC3B, WIPI-1, VMP-1, pJNK, cleavage of caspases, and poly (ADP-ribose) polymerase (PARP)	[39]
Lung cancer		Ground stems extract (*C. odorata*)	NCI-H460	-	Exhibits cytotoxic effects Inhibits cell proliferation Apoptosis induction	Causes cell cycle arrest at the S phase and induces apoptosis.	[31]
		Commercial	A-549	-	Apoptosis induction	Hsp90 inhibition regulates the PI3K/AKT signaling pathway, inhibits autophagy, and induces apoptosis in cells	[40]
		Commercial nano-encapsulated	NCI-H292	-	Inhibits cell proliferation Apoptosis induction	Enhances anti-proliferative effects against cells through *p53*-initiated, *Bax*-associated, caspase-dependent activation of apoptosis	[41]
Brain cancer		Commercial	U-251 MG	-	Effects on tumor invasion and angiogenesis	Inhibits proliferation, migration and invasive potential of the cells through inhibition of matrix metallopeptidase 9 (MMP-9), focal adhesion kinase (FAK), and Rho-associated protein kinase 1 (ROCK-1)	[42]
Leukemia		Flower extract (*Carapa guianensis*)	P388 HL60	-	Exhibits cytotoxic effects	-	[43]
		Seed extract (*A. indica*)	HL60	-	Exhibits cytotoxic effects	-	[32,33]
Skin cancer		Ground stem extract (*C. odorata*)	A375-C5	-	Exhibits cytotoxic effects	-	[31]
		Seed extract (*A. indica*)	B16	-	Exhibits cytotoxic effects Inhibits activity	-	[32,44]
Cancer stem cells		Commercial	NTERA-2	-	Inhibits cell proliferation Apoptosis induction	Enhances anti-proliferative effect in cells; inhibits Hsp90 and *survivin*; upregulates *Bax* and *p53*; induces DNA fragmentation and increases caspase-3, caspase-7 activity	[45]

## Abbreviations

A375-C5	Human skin cancer cell line
A-549	Human lung cancer cell line
A2870	Human ovarian cancer cell line
AD	Alzheimer’s disease
AKT	Serine/threonine kinase
AR	Androgen receptor
ARase	Aldose reductase
ATP	Adenosine triphosphate
ATPase	Adenosine triphosphatases
AZ521	Human gastric cancer cell line
B16	Mouse skin cancer cell line
Bax	B-cell lymphoma-2 protein associated X protein
Bcl2	B-cell lymphoma 2 protein
C30	Human ovarian cancer cell line
CaCo-2	Human colon cancer
Cdc37	Hsp 90 co-chaperone encoded by the CDC37 gene
CDK1	Cyclin dependent kinase 1
CHK1	Checkpoint kinase 1
CP70	Human ovarian cancer cell line
CSCs	Cancer stem cells
D-GalN	D-galactosamine
DM	Diabetes mellitus
DMBA	7,12-Dimethylbenz[a]anthracene
DNA	Dezoxiribonucleic acid
EC_50_	Half maximal effective concentration
ED_50_	Median effective dose
EMT	Epithelial-to-mesenchymal transition
FAK	Focal adhesion kinase
GI_50_	Median growth inhibition concentration
GPK	Guinea-pig ear epidermis cells
HBP	Hamster buccal pouch
HeLa	Human cervical cancer cell line
HL60	Human promyelocytic leukemia cell line
HIF1α	Hypoxia inducible factor 1 alpha
HME	Human mammary epithelial cell line
HPA	Human pancreatic α-amylase
HPAC	Human pancreatic cancer cell line
Hs578Bst	Human breast normal cell line
HSF1	Heat shock factor 1
Hsp	Heat shock protein
Hs578T	Human breast cancer cell line
hTERT-HPNE	Human pancreatic normal cell line
IC_50_	Half maximal inhibitory concentration
ID8	Mouse ovarian cancer cell line
ID8TaxR	Raclitaxel-resistant mouse ovarian cancer cell lines
IKK	Inhibitory kappa B kinase
LC_50_	Half maximal lethal concentration
LD_50_	Median lethal dose
LNCaP	Human prostate cancer cell line
LPS	Lipopolysaccharide
MCF7	Human breast cancer cell line
MDA-MB-231	Human breast cancer cell line
MDA-MB-453	Human breast cancer cell line
MIAPaCa-2	Human pancreatic cell line
MIC	Minimum inhibitory concentration
miR-21	MicroRNA-21
mRNA	Messenger ribonucleic acid
MMP-9	Matrix metallopeptidase 9
MRC-5	Human lung normal cell line
mTOR	Mammalian target of rapamycin
NCI-H292	Human lung cancer cell line
NCI-H460	Human lung cancer cell line
NF-kB	Nuclear factor kappa B
Nrf2	Nuclear factor erythroid 2-related factor 2
NO	Nitric oxide
NTERA-2	Human pluripotent embryonal cancer cell line
OVCAR4	Human ovarian cancer cell line
OVCAR8	Human ovarian cancer cell line
P338	Menogaril-resistant mouse leukaemia cell line
PANC-1	Human pancreatic cell line
PARP	Poly (ADP-ribose) polymerase
PfPK5	*Plasmodium falciparum* protein kinase 5
PHD2	Prolyl hydroxylase domain protein 2
PI3K	Phosphoinositide 3-kinases
PLK1	Polo-like kinase 1
PD	Parkinson’s disease
ROCK-1	Rho-associated protein kinase 1
SCC131	Human oral squamous cancer cell
SH-SY5Y	Human neuronal cells
SK-BR-3	Human breast cancer cell line
SKOV3	Human ovarian cancer cell line
STS	Standard target species
TLR	Toll-like receptor
U-251 MG	Human brain cancer cell line
WHO	World Health Organization
